# Commandeered Hospitals: the Question of Compensation

**Published:** 1917-11-24

**Authors:** 


### COMMANDEERED HOSPITALS.

### The Question of Compensation.

In reference to a proposal to acquire the new Robroyston Hospital for military uses, the Town Clerk of Glasgow has prepared for the Corporation a special report with reference to the powers of the military authorities, who, he points out, may acquire the hospital compulsorily under the Defence of the Realm Regulations.

There are no specific provisions either in the Defence of the Realm Acts or the Regulations for the payment of compensation for land or buildings taken, but a Commission had been appointed to deal with claims, adopting the following principle: "Ascertaining the amount of such loss and damage and paying the same out of public funds."

The War Department deals with some claims itself instead of referring them to the Commission, and this was done in the case of the ground occupied by Bellahouston Red Cross Hospital. There the competent military authority took possession. In the notice under which possession was taken it was stated that the War Department would pay rent under any existing agreement up to the date of the notice, and that thereafter, possession being under the Defence of the Realm Regulations, no legal liability for rent or other form of compensation would be incurred, the question of compensation being in the discretion of the War Department and of an *ex gratia* nature. As a result of negotiation, the War Department agreed to pay the amount of the grazing rent—namely, £17.

In the case of Poor-Law institutions occupied for military purposes the matter of compensation was dealt with in a memorandum issued by the Local Government Board, in terms of which the Army Council, while not paying a rent or making any contribution in respect of existing capital charges, will reimburse to the Poor-Law authorities the whole of their necessary and authorised expenditure upon the occupied premises during the period of the military occupation, including the remuneration of all officers and servants employed during the military occupation in or about the premises. On the other hand, an account will be required showing how the cost of the inmates displaced by the military occupation has been affected by their displacement, and the difference between the actual cost to the Poor-Law authority of those inmates and the cost which would have been incurred had no military occupation taken place will be debited, or credited, as the case may be, to the Army Council.

The basis of compensation set forth in this memorandum would appear only to be applicable to the case of an institution occupied at the time it was taken over by the military authorities, and might not apply to Robroyston Hospital, which has never been occupied. This is the view taken by the military authorities, their contention being that, as the premises have not yet been brought into use, no additional expenditure will be entailed, and any question of a credit or debit, in respect of a variation in the cost of maintenance, will not arise.

In this connection it may be mentioned that, as a result of the proposed military occupation of the hospital, it is proposed to provide other accommodation, involving a large capital expenditure—namely, the extension of Knightswood Hospital and Bellefield Sanatorium, and the erection of a sanatorium at Southfield, and the cost of these is estimated at £193,000, exclusive of costs already incurred.

The foregoing principles of compensation apply to land and buildings occupied by the military during the period of the war. A different arrangement comes into operation on the termination of the war. By the Defence of the Realm (Acquisition of Land) Act, 1916, the Government may continue in possession after the termination of the war for two years and, with the consent of the Railway and Canal Commission, for a further period of three years; but in that event rent is to be paid for the land for the period of possession after the termination of the war.

